# Comprehensive Management of Upper Tract Urothelial Carcinoma

**DOI:** 10.1155/2009/656521

**Published:** 2008-12-10

**Authors:** Georgios Koukourakis, Georgios Zacharias, Michael Koukourakis, Kiriaki Pistevou-Gobaki, Christos Papaloukas, Athanasios Kostakopoulos, Vassilios Kouloulias

**Affiliations:** ^1^Radiation Therapy Unit, 2nd Department of Radiology, Athens University Medical School, Attikon University Hospital, Rimini 1 Street, Haidari, 12462 Athens, Greece; ^2^Section of Pathology, Policlinic of Athens, Piraeus 5 Street, 11474 Athens, Greece; ^3^Radiation Therapy Unit, University Hospital of Thrace, 68100 Alexandroupolis, Greece; ^4^Radiation-Oncology Department, AHEPA University Hospital of Thessalonica, 54301 Thessalonica, Greece; ^5^Section of Urology, University Hospital of Athens, 10029 Athens, Greece

## Abstract

Urothelial carcinoma of the upper urinary tract represents only 5% of all urothelial cancers. The 5-year cancer-specific survival in the United States is roughly 75% with grade and stage being the most powerful predictors of survival. Nephroureterectomy with excision of the ipsilateral ureteral orifice and bladder cuff en bloc remains the gold standard treatment of the upper urinary tract urothelial cancers, while endoscopic and laparoscopic approaches are rapidly evolving as reasonable alternatives of care depending on grade and stage of disease. Several controversies remain in their management, including a selection of endoscopic versus laparoscopic approaches, management strategies on the distal ureter, the role of lymphadenectomy, and the value of chemotherapy in upper tract disease. Aims of this paper are to critically review the management of such tumors, including endoscopic management, laparoscopic nephroureterectomy and management of the distal ureter, the role of lymphadenectomy, and the emerging role of chemotherapy in their treatment.

## 1. Introduction

Primary urothelial carcinoma of the upper tract is a rare urological disease and has a propensity for multifocality, local recurrence, and development of metastases. Almost 5% of all urothelial neoplasms occur in the kidney and ureters. The vast majority of upper tract tumors arise in the kidney, comprising 4% to 15% of all primary kidney neoplasms in the United States, whereas ureteral tumors represent only 1% [1].

As a result, urothelial carcinoma of the bladder has been examined to a greater extent than urothelial tumors elsewhere.

The main treatment for patients with upper tract urothelial neoplasms and a normal contralateral kidney is a complete nephroureterectomy with removal of a cuff of urinary bladder. Due to the high rate of ureteral stump recurrence, which has been reported to be between 30% and 75%, it is important to complete the nephroureterectomy with a cuff of urinary bladder [2–10]. Hall et al. [11] reported in one of the largest series in the literature on 252 patients who were treated for upper tract urothelial tumors with a median follow-up of 64 months. One hundred ninety-four (76.6%) patients underwent open radical nephroureterectomy with removal of bladder cuff, 42 (16.7%) patients underwent parenchymal-sparing surgery, 14 (5.6%) patients underwent nephrectomy alone, and 2 (0.8%) had exploration only for nonresectable disease. Overall, patients undergoing parenchymal-sparing surgery had a lower actuarial 5-year disease-free survival rate than those treated with initial aggressive surgical resection (23% versus 45%, *P* < .0009). Patients with grades 1 and 2 tumors were equally distributed in these 2 groups. This study supported the use of aggressive open surgical resection for initial treatment of upper tract urothelial tumors, with a 5-year disease-free survival rate of 45%.

Nevertheless, the gold standard of open radical nephroureterectomy with resection of a bladder cuff is being challenged by minimally invasive approaches to the managing of upper tract transitional cell carcinoma (TCC). For upper tract urothelial carcinoma, laparoscopic nephroureterectomy has been used as an alternative to an open procedure. Since the first laparoscopic nephroureterectomy, performed by Clayman in May 1991 at Washington University (St. Louis, Mo, USA), numerous reports regarding the safety and efficacy of that procedure have been published [12, 14–23]. This paper will cover the therapeutic approaches to upper tract TCC, including laparoscopic nephroureterectomy, endoscopic approaches, and the prognostic value of lymphadenectomy in patients with muscle invasion. Topical immunotherapy, adjuvant chemotherapy, and adjuvant radiation therapy will also be discussed.

## 2. Surgical Treatment

### 2.1. Laparoscopic Treatment

Recently, Gill et al. published on their experience of 42 patients who underwent laparoscopic retroperitoneal nephroureterectomy with a mean follow-up of 11.1 months [12]. The distal ureter was treated with a combination of laparoscopic and endoscopic transvesical approaches [23]. A comparable research was performed between those patients and another 35, who underwent open nephroureterectomy at their department. In the laparoscopic group, the blood lost was significantly less (242 versus 696 mL). Postsurgically, patients in the laparoscopic group had a significantly more rapid recommencement of ambulation (1.4 versus 2.5 days), oral intake (1.6 versus 3.2 days), shorter hospital stay (2.3 versus 6.6 days), decreased analgesic necessities (26 mg morphine sulfate equivalent versus 228 mg), and a more rapid period of recovery (8 versus 14.1 weeks). Complications occurred in 5 (12%) and 10 (29%) patients in the laparoscopic and open groups, respectively. These complications integrated 1 renal vein injury, 1 patient with fluid extravasations from mobilization of the bladder cuff, and 3 patients with atelectasis in the laparoscopic group. The open group had 4 patients with atelectasis, 5 patients with postoperative ileus, and 1 patient with a pneumothorax. Two cases required an open conversion because of a renal injury and an elective conversion secondary to local tumor infiltration with obliteration of tissue planes near the hilum.

**Figure 1:**
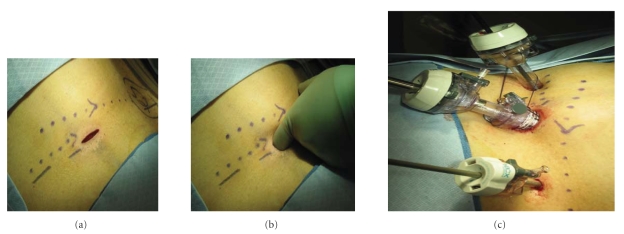
(a) Access for a right retroperitoneal laparoscopic radical nephrectomy (LRN). A 10- to 15-mm incision is made below and medial to the tip of the 12th rib. (b) The flank muscles are pierced with a blunt-tipped instrument followed by finger dissection and development of the retroperitoneum space to permit trocar placement. (c) Trocar placement for a right retroperitoneal LRN.

**Figure 2:**
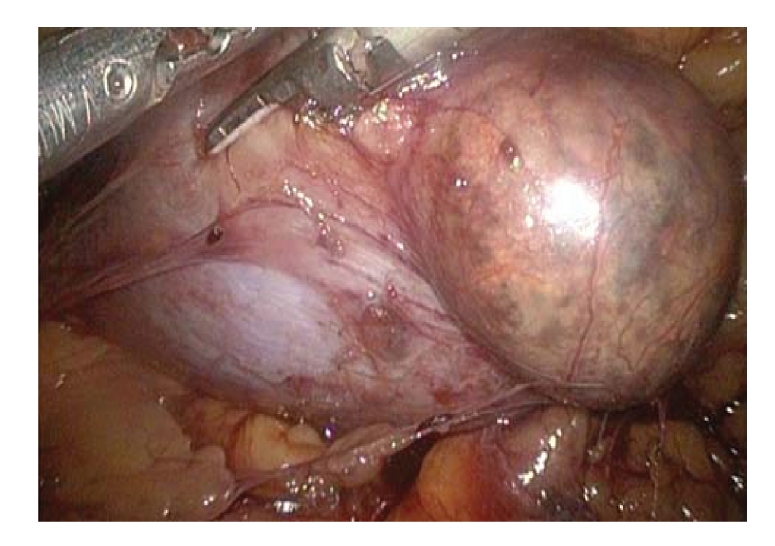
Retroperitoneal laparoscopic left partial nephrectomy.

The mean pathologic grade was 2.3 for both of the groups, with the laparoscopic group having 9, 10, and 23 patients with grades 1, 2, and 3 tumors and the open group having 6, 10, and 16 patients with grades 1, 2, and 3 tumors, respectively. Surgical margins were positive in 3 (7%) patients in the laparoscopic group and 5 (15%) patients in the open group. All 3 patients in the laparoscopic group received systemic chemotherapy postoperatively, and pulmonary metastases developed in 1 patient during follow-up. For comparable stage and grade of primary tumor, the negative surgical margin rate was similar between the 2 groups. The two groups of laparoscopic and open surgeries have no difference as regarding the bladder recurrence (23 versus 37%), retroperitoneal or port site/incisional recurrence (0 versus 0%), or distant metastases (8,6 versus 13%). There was no difference in either cancer-specific survival (97% versus 87%) or crude survival (97% versus 94%) after adjusting for the shorter follow-up period (11 versus 34 months) between the laparoscopic and open groups, respectively, during follow-up. Mortality occurred in 2 patients (6%) of the laparoscopic group and in 6 of the open group (30%). The authors cannot estimate whether these mortality rates are significantly different or equivalent [12]. The results of the trails that compare laparoscopic treatment with open surgery are summarized in Table 1. The techniques of laparoscopic retroperitoneal nephroureterectomy and partial nephrectomy are shown in Figures 1(a), 1(b), and 1(c) and in Figure 2, respectively. Patients are placed in the full flank position. Usually, the operating table is flexed and the kidney rest is elevated, thereby increasing the space between the iliac crest and the lower ribs. Retroperitoneal access is obtained through a small 10- to 15-mm incision just below and medial to the tip of the 12th rib (Figure 1(a)). The muscle and fascia are separated using a blunt instrument that allows one finger to develop a working space posterior to the kidney above the psoas muscle (Figure 1(b)). Often the lower pole of the kidney is immediately palpated. The colon is separated away from the anterolateral abdominal wall with the index finger. Additional trocars are placed below the 12th rib just above the psoas muscle posteriorly, and then more anteriorly through the lateral abdominal wall under direct vision using the laparoscope (Figure 1(c)). Laparoscopic partial nephrectomy is ideal for a patient with an exophytic, small, and peripherally located renal tumor (Figure 2). 

The literature research discovered similar effects in transperitoneal laparoscopic nephroureterectomy [13, 24]. The familiarity with anatomic landmarks and a larger working space are some advantages of transperitoneal approach compared to retroperitoneal one. The retroperitoneal approach, however, has distinct advantages, for example, permittance of early control of the renal artery and vein, no manipulation of the bowel leading to less incidence of ileus and possibly a shorter hospital stay, and confinement of possible urinomas or seromas to the retroperitoneal space [12, 25].

### 2.2. Hand-Assisted Laparoscopic Nephroureterectomy

**Figure 3:**
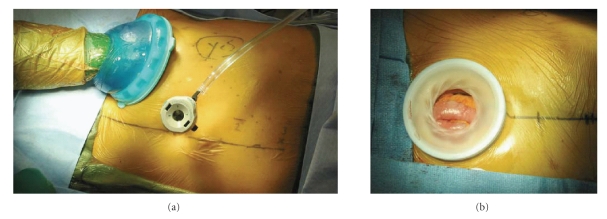
(a) Right lower quadrant hand-port placement for hand-assisted right radical nephrectomy, and (b) Lower midline hand-port placement for hand-assisted right radical nephrectomy.

Apart from standard laparoscopic nephroureterectomy among urologists, hand-assisted laparoscopic nephroureterectomy is also an acceptable technique [18, 20]. Patient preparation and positioning is identical to that described for transperitoneal LRN (laparoscopic nephroureterectomy). The hand-assisted LRN technique usually begins with a 6 cm to 8 cm incision for hand-port placement through a lower quadrant Gibson-type incision (Figure 3(a)) or through the lower midline abdomen (Figure 3(b)). The Kawauchi et al.'s [18] experience was described in 34 consecutive patients who underwent hand-assisted laparoscopic nephroureterectomy using a Lap Disc (Hakko Shoji, Tokyo, Japan). Those 34 patients were compared with the previous group of 34 patients who underwent open nephroureterectomy. Mean follow-up was 13.1 months in the hand-assisted group and 48.8 months in the open group [18]. In the hand-assisted group, there was observed a similar operative time (233 versus 236 minutes), decreased analgesia frequency (2.1 versus 4.1 days), decreased blood loss (236 versus 427 mL), quicker return to ambulation (1.5 versus 2.5 days), and shorter hospital stay (13 versus 21.1 days). In the Japanese series compared with the American ones, the lengthy stay is a social issue and not reflective of actual patient recovery. There were 4 (12%) complications in both groups, with 1 open conversion in the hand-assisted group. The 4 complications in the hand-assisted group include 1 conversion due to bleeding from the left adrenal gland, 2 wound infections, and 1 pulmonary infarction in a patient who recovered with conservative treatment [18]. As regarding the histological tumor grade, the pathologic studies have revealed that in the hand-assisted group there were 5, 20, and 9 patients with grades 1, 2, and 3 tumors, whereas in the open group there were 4, 17, and 13 patients with grades 1, 2, and 3 tumors, respectively. Recurrence rate was 12% (4 patients) in the hand-assisted group, with a mean time to recurrence of 9.5 months. Patients in the open group had a longer mean time to recurrence at 14.4 months, with a 47% (16 patients) recurrence rate [18].

Seifman et al. [26] completed a prospective study comparing 16 patients (mean follow-up, 19 months) who underwent hand-assisted laparoscopic nephroureterectomy to 11 patients (mean follow-up 16 months) who underwent the open technique. Despite the fact that the operative time was longer in the hand-assisted group (320 versus 199 minutes), there was a decrease in the length of hospital stay (3.9 versus 5.2 days), time to oral intake (33 versus 38 hours), analgesic requirements (20 versus 31 tablets), and return to normal activity (18 versus 38 days). Tumor recurrence appeared in 3 of 16 laparoscopic cases and in 7 of 11 open cases. However, the open series had a higher number of patients with grade 3 (6 of 11) and T3 disease (5 of 11) compared with the laparoscopic group (5 of 16 with grade 3, 3 of 16 with T3 disease).

Landman et al. [20] compared 16 patients who underwent hand-assisted laparoscopic nephroureterectomy to 11 patients who underwent a standard one. In the standard group, the mean follow-up was 27.4 months, whereas in the hand-assisted group it was 9.6 months. Compared with the standard technique, patients who underwent the hand-assisted technique had a decreased operative time (4.4 versus 5.3 hours), similar blood loss (201 versus 190 mL), longer time to oral intake (20 versus 13 hours), similar analgesic use (33 versus 29.3 mg of morphine), longer hospital stay (4.5 versus 3.3 days), and longer time to complete recovery (8 versus 5.2 weeks).

Complications occurred in 5 patients in both groups, with 1 open conversion in the hand-assisted group due to failure to progress. Myocardial infarction and respiratory failure was the cause of death postoperatively for a patient in the hand-assisted group. Pathologic stage and grade were similar in the 2 groups, with the majority of patients having low-grade and low-stage tumors. Metastatic disease developed in 3 out of 15 hand-assisted cases and in 2 patients of the standard group [20].

The results of the trails that compare hand-assisted laparoscopic nephroureterectomy with other techniques are summarized in Table 2. Thereafter, these studies sustain the utility of both hand-assisted and pure laparoscopic techniques for nephroureterectomy. The hand-assisted technique has the advantage of the tactile sensation and blunt-manual dissection. The probability of cancer control is similar to open techniques. However, due to the fact that most of the studies were current, long-term action—a five-year period—is required for definitive results.

### 2.3. Robotic-Assisted Laparoscopic Management of Upper Urothelial Tract TCC

The daVinci robotic surgical system has revolutionized minimally invasive urologic laparoscopy as applied to prostatectomy. By providing a three-dimensional operating environment and instrumentation with two additional degrees of freedom, the daVinci surgical system appears to have dramatically reduced the learning curve for complex-laparoscopic procedures. Even laparoscopically inexperienced open surgeons can become remarkably talented in a technically challenging procedure such as robotic radical prostatectomy in as few as 12 patients [27].

Since there are not any studies regarding the robotic-assisted laparoscopic management of upper urothelial tract carcinoma, we analyze the papers of robot-assisted laparoscopic partial nephrectomy and we believe that the technique will be soon applicable for small lesions of renal pelvis and upper ureter.

The first series of robot-assisted laparoscopic partial nephrectomy (RLPN) for small renal masses was reported by Gettman et al. [28]. Since then, there have been five other reports, three of which detail the New York University experience [29–33]. The results of the last trail conducted by Deane et al. [33] as regarding the mean tumor size, the mean total procedure time, the mean estimated blood loss, and the mean warm ischemia time are similar to those analyzed in the previously reported series, and by comparing them with laparoscopic partial nephrectomy (LPN), there were no differences. Moreover, in this cohort there were no conversions, while in the New York University series, among 12 patients, there were two conversions: one to a hand-assisted approach and one to an open approach [30]. In the series of Kaul et al. [32], like in that of Deane et al. [33], there were no conversions; however, a patient had urgent re-exploration and nephrectomy. These studies are summarized in Table 3.

RLPN using the daVinci surgical system can be performed by a fellowship of trained urologic oncologists with extensive experience in robotic radical prostatectomy; early results mirror those achieved by experienced laparoscopic surgeons performing standard LPN. These results can further support the assumption of introducing a robotic interface which provides surgeons with extensive experience in open and other robotic procedures (in this instance, open partial nephrectomy and robot-assisted radical prostatectomy), with the successful incorporation of advanced robotic procedures, such as partial nephrectomy, into their clinical practice.

### 2.4. Management of Distal Ureter and Bladder Cuff

While there is a negligible disagreement about the role of laparoscopic nephroureterectomy, the management of the distal ureter and bladder cuff with laparoscopy varies among the surgeons. Techniques include (1) open excision, (2) transvesical laparoscopic detachment and ligation technique, (3) laparoscopic stapling of the distal ureter and bladder, and (4) the “pluck” technique. Steinberg and Matin have recently reviewed these techniques [21].

#### 2.4.1. Open Technique

An open technique involves initial dissection of the renal unit laparoscopically. After its completion, the ureter is clipped but not ligated to avert potential downstream seeding of tumor cells. Once the laparoscopic ports are separated, either a midline, Gibson, or Pfannenstiel incision is performed. The distal ureter is identified and dissected towards the bladder. The specimen is then isolated en bloc with a border of bladder cuff. The bladder may be opened and the ureter dissected intravesically and extravesically, or secured and the full dissection performed extravesically.

Matsui et al. [22] reported their results in 17 patients who underwent laparoscopic nephroureterectomy using an open technique to remove the distal ureter and bladder cuff. A comparison with another 17-patient group who underwent standard nephroureterectomy was performed. The mean follow-up was 8.8 months in the laparoscopic group and 23.0 months in the standard group. Patients who were in high risk and had good performance status had received adjuvant chemotherapy postoperatively. In the laparoscopic group, 1, 6, and 10 patients had grades 1, 2, and 3 diseases on final pathologic examination. The standard group had 0, 6, and 11 patients with grades 1, 2, and 3 diseases on final pathologic examination. T3 disease was found in 5 patients in both groups with the rest of patients having T2 or lower disease. Three patients in the laparoscopic group and four in the standard group, respectively, had received adjuvant chemotherapy. The recurrence was observed only in a patient in the laparoscopic group, in comparison to six in the standard group, but that could be attributed to the shorter follow-up of the laparoscopic group. After adjusting to that difference in follow-up, there were no significant differences in the disease-free survival rate between the 2 groups [22].

Klingler et al. [16] also reported on 19 patients who underwent laparoscopic nephroureterectomy; mean follow-up 22.1 months, with an open approach to remove the distal ureter and bladder cuff. The comparison was made to 15 patients who underwent standard nephroureterectomy, mean follow-up 23.1 months. According to the T stage, there were 12 patients with T1 versus 10, 2 patients with T2 versus 2, and 5 with T3 versus 3 in the laparoscopic and standard groups, respectively. Tumor recurrence was observed in a patient in both groups who had grade 3 and T3 disease. This study also concluded that the risk for tumor recurrence and cancer control rates was similar between the standard technique and the laparoscopic group with an open technique of handling the distal ureter and bladder cuff.

#### 2.4.2. Transvesical Laparoscopic Technique

Gill et al. [23] have used a transvesical laparoscopic technique to remove the distal ureter and bladder cuff. That was performed by using 2 needlescopic ports placed suprapubically into the bladder under cystoscopic guidance. The patient was repositioned into the dorsal lithotomy position before placing the bladder ports. A ureteral catheter was then placed in the ipsilateral orifice through an endoloop that was passed through the laparoscopic bladder ports. A grasper was used to tent the ureter anteriorly and a Collins knife to dissect the bladder cuff and ureter. The intramural ureter and bladder cuff were completely detached en bloc from the bladder. The dissection continued with the Collins knife into the pelvic extraperitoneal fatty tissues.

Gill et al. [12] compared 42 patients who underwent that technique to 35 patients who underwent the standard open nephroureterectomy. That study-case was discussed earlier in this study and as a conclusion the patients had comparable cancer-specific survival and tumor recurrence. The follow-up, however, was shorter for patients who underwent the transvesical laparoscopic technique.

Stifelman et al. [34] have also reported using a combined transvesical laparoscopic and endourologic technique on 22 patients with an average follow-up of 13 months. The pathologic examination has revealed that 3, 10, and 9 patients had grades 1, 2, and 3 tumors. Five lesions were Ta, 8 were T1, 2 were T2, and 7 were T3 disease. In all cases, the margins were negative. Disease recurrence was observed in six patients: four with low grade, low-stage bladder tumors, not involving the resection site, and two with grade III T3 tumors who presented later with metastatic lesions. All patients were alive at 18 months. This technique simulates established open principles for upper tract urothelial tumors. Potential criticisms of this technique are the risk of fluid extravasation and subsequent potential tumor seeding. This is minimized, however, by continuous suction from the transvesical ports. Furthermore, a meta-analysis of the literature reveals no reports of tumor seeding in over 50 patients to date [12, 21, 23, 34, 35]. In cases in which tumor is presented in the distal and intramural ureter, active bladder disease exists, and in patients who have received prior pelvic radiation therapy this technique is contraindicated.

#### 2.4.3. Laparoscopic Stapling Technique

Laparoscopic stapling of the distal ureter and bladder cuff has been combined with cystoscopic unroofing [13, 21]. With this procedure, ureteral unroofing is performed initially via cystoscopy and placement of a balloon catheter in the intramural ureter. The distal ureter and bladder cuff are then stapled laparoscopically during the distal dissection, using an Endo-GIA (US Surgical, Norwalk, Conn, USA) stapler.

Shalhav et al. [13] reported their experience using the laparoscopic stapling technique in 25 patients who underwent laparoscopic nephroureterectomy and compared them with 17 patients who underwent open radical nephroureterectomy. A patient in the laparoscopic group underwent the “pluck” technique, which will be discussed later in this review. Mean follow-up was shorter in the laparoscopic group (24 versus 43 months). Thirteen patients in both groups had grade 2 disease or greater. Distal metastases developed in 4 patients (31%) in the laparoscopic group and 3 patients (23%) in the open group. In the laparoscopic group, local recurrence rate was lower, 3 versus 7, but this could be attributed to a shorter follow-up. All patients with recurrence in the laparoscopic group had tumors that recurred in the bladder and were treated with transurethral resection. The authors argue in this series that the stapling technique minimizes the risk of tumor spillage, since the bladder cuff just caudal to the ureter is secured and occluded with six rows of titanium staples before it is incised.

Yoshino et al. [37] also reported their experience with 23 patients using flexible endoscopic gastrointestinal automatic stapler (Ethicon Endosurgery, Cincinnati, Ohio, USA) in their laparoscopic series. At a mean follow-up period of 15 months, 4 patients had bladder recurrence which was successfully treated by transurethral resection. Three of those patients had no evidence of disease at greater than a 20-month-follow-up, whereas 1 died of other medical comorbidities.

While the previous studies support the use of the stapling technique for distal ureteral and bladder cuff management, Matin and Gill [35] evaluated outcome and patterns of recurrence based on the form of bladder cuff control. They concluded that positive margins were higher with a laparoscopic stapling approach than either the open or the transvesical technique. Additionally, the stabling technique was associated with poorer recurrence-free survival.

The theoretical risk of stone formation, secondary to the migration of staples into the bladder mucosa, could be an additional animadversion of this technique. Chandhoke et al. [38] reported that there was neither stone formation nor visible staples in the bladder after using the stapling technique. A recent case report revealed the presence of a nearly complete intravesical titanium staple line on surveillance cystoscopy at a follow-up of 6 months [39]. However, there was no identifiable encrustation in that patient, and a successful transurethral resection of the staple line was performed without sequela.

#### 2.4.4. The “Pluck” Technique

The “pluck” technique involves an aggressive transurethral resection of the ipsilateral ureteral orifice with a simultaneous “plucking” of the distal ureteral during the laparoscopic procedure. Before the resection of the renal unit and ureter, this resection is performed initially via a resectoscope. McNeil et al. [36] treated 25 patients using that technique and compared them with 42 patients who underwent open nephroureterectomy. In the laparoscopic group, the follow-up was shorter mean 32.9 versus 42.3 months. According to tumor grade, in the laparoscopic group, there were 4, 6, and 9 patients with grades 1 and 2, while in the open group there were 2, 8, and 6 patients with grade 3. Pathologic examination also revealed T1, T2, and T3 diseases in 0, 1, and 9 patients in the laparoscopic group and 0, 3, and 6 patients in the open group. Four deaths in the laparoscopic group and nine in the open group were observed. The authors concluded that there was no increase in local recurrence within the laparoscopic group during the follow-up, but the exact incidence of recurrence was not reported. The studies that compare the techniques of the management of distal ureter and bladder cuff with other methods of treatment are summarized in Table 4.

The major criticism of that technique is tumor seeding and the potential to leave behind a segment of an incompletely resected ureter [21, 40–42]. Arango et al. [40] described a case of a fatal recurrence at the resection site after endoscopic resection of the intramural ureter. The patient had stage 1 grade 2 transitional cell carcinoma with a normal lower ureter and bladder. Nevertheless, seven months later, the patient presented with pelvic pain and urgency. Computed tomography revealed a large vesical mass at the site of the resected lower ureter. The biopsy showed a grade 3 tumor stage IV and the patient underwent salvage cystectomy with adjuvant chemotherapy. Three months after cystectomy the patient died. The exact incidence of tumor seeding is unknown and difficult to assess. On the other hand, the theoretical potential combined with the above reports has led some authors to abandon this technique [40–42].

To summarize, laparoscopic nephroureterectomy with open distal ureterectomy is a safe and acceptable alternative to open nephroureterectomy. Cancer control rates seem to be similar with superior convalescence. In terms of managing the distal ureter and bladder cuff, the open technique is the most efficacious so as to achieve negative margins and decreased risk of cancer seeding. However, because of the relatively small series in the literature (due to the low incidence and prevalence of the disease) and because most literature is fairly recent (due to recent advances), long-term follow-up and larger series are necessary to assess cancer-specific survival and recurrence rates.

## 3. Endoscopic Management

Generally, recommendations for endoscopic management of upper-tract TCC include patients with anatomic or functional solitary kidneys, bilateral upper-tract TCC, base line renal insufficiency, or significant comorbid diseases that preclude abdominal surgery [43]. Patients with a normal contralateral kidney who have small, low-grade lesions can also be reasonable candidates for conservative management [44]. Endoscopic treatment of the upper urinary tract can be performed via either a retrograde ureteroscopic or a percutaneous antegrade approach.

As regarding the retrograde ureteroscopic, an approach can be used for low-volume ureteral and renal pelvic tumors. Proximal ureter and renal pelvic lesions require flexible ureteroscopes, while tumors localized in the intramural and distal ureter are best managed by rigid ureteroscopy [45]. Low morbidity in association with maintenance of urothelial integrity is the principal advantage of retrograde endoscopy [43, 45, 46]. This technique is limited, however, by the size of instruments that can be accommodated in the ureter, which in turn limits the size of tumor that can be adequately treated. Some portions of the upper urinary tract, such as the lower pole calyces, are less accessible by a retrograde approach. Furthermore, retrograde ureteroscopy differs in patients who have undergone a prior urinary diversion.

An initial biopsy of the lesion is required for the ureteroscopic method followed by a debulkment to its base using cold-cup forceps (3 Fr or 5 Fr) or a stone basket (1.9 Fr or 2.4 Fr) [45]. Due to the fact that the wall of the proximal ureter and renal pelvis is thin, no attempt should be made to resect these regions deeply. The base of the lesion is subsequently addressed by monopolar electrocautery or laser ablation (neodymium: yttriumaluminum-garnet [Nd:YAG] or holmium [Ho]: YAG laser) [47]. With a tissue penetration of less than 0.5 mm, the Ho:YAG laser is well suited for use in the ureter, allowing for excellent hemostasis with minimal transmural thermal damage. Conversely, the Nd:YAG laser has a deeper penetration (5-6 mm) making it better suited for coagulative necrosis of large lesions, particularly in the renal pelvis [46].

Ureteral perforation and postoperative strictures are the principal complications associated with retrograde ureteroscopy. The incidence of perforation in most series is below 10% and is readily managed by ureteral stenting or percutaneous nephrostomy drainage [48, 49]. The reported stricture rate following retrograde management of upper-tract TCC ranges from 4.9% to 13.6% [48–50]. Literature data indicate that a lower incidence of strictures is associated with lesions managed by laser ablation, rather than with electrocoagulation [51]. Most postoperative strictures are successfully managed by endoscopic stenting, laser incision, or balloon dilatation. Ultimately, all ureteroscopic interventions should be followed with short-term ureteral stenting to prevent postoperative obstructive sequelae.

Nevertheless, being more invasive than retrograde ureteroscopy, the percutaneous antegrade approach is preferred in larger tumors of the renal pelvis and proximal ureter. Antegrade nephroscopy offers better visualization of the renal pelvis whereas accommodating larger caliber working instruments, being able to handle a larger tumor burden. The percutaneous approach also allows for superior access to the lower pole calyces, as well as to renal units with complicated calyceal anatomy. The principal disadvantage of this approach is violation of urothelial integrity with reports of tumor seeding of nonurothelial surfaces around the kidney or in the nephrostomy tract [52, 53]. Larger series, however, fail to note such tract recurrences, confirming that this phenomenon is uncommon [54–56].

After a percutaneous tract that can accommodate a 30 Fr access sheath is subsequently established, the lesion is initially biopsied and consequently debulked. Due to the larger access tract, antegrade techniques permit the use of cold-cup biopsy forceps through a standard nephroscope or a cutting loop from a resectoscope. The base of the lesion is resected and sent separately for staging purposes, and haemostasis is achieved by electrocautery or laser ablation as previously described. The established nephrostomy tract can be maintained, allowing for repeated treatment or administration of topical adjuvant therapy [45, 46].

Away from tumor tract seeding, complications of percutaneous management of upper-tract TCC are similar to those of percutaneous stone procedures and include bleeding, infection, electrolyte abnormalities, adjacent organ injury, and pleural injury [45, 46].

The safety and efficacy of ureteroscopic management of upper-tract TCC are confirmed by multiple studies. In 1997, Tawfiek and Bagley reported on the outcomes of 205 patients summarized from 14 modern series and found a recurrence rate of 33% for 61 renal pelvic tumors and 31% for 144 ureteral tumors [57]. More recent reviews demonstrate similar findings, with recurrence rates ranging from 31% to 65% and disease-free rates of 35% to 86% [47, 49, 58–62]. The bladder was the most frequent site of recurrence in these series. Tumor recurrence was most dependent upon pathologic grade with recurrence rates of 25% for grade I tumors and almost 50% for higher-grade lesions [48]. It is important to note that initial endoscopic management does not predict a worse outcome if disease progression occurs. Boorjian and colleagues reported that ureteroscopic tumor ablation before nephroureterectomy did not adversely affect postoperative disease status [63].

As regarding the percutaneous approaches, they have promising results when taking into consideration that these lesions are more substantial than those managed by retrograde ureteroscopy. Okada et al. performed a review in 84 patients and found an overall recurrence rate of 27%, with tumor grade strongly predicting outcomes [64]. Additionally, Rouprêt et al. reported a similar recurrence rate of approximately 30%, with 5-year disease specific survival of almost 80% [65]. Moreover, Lee et al. reviewed their 13-year experience with percutaneous management of upper-tract TCC patients and found no significant differences in overall survival compared with those patients who underwent a nephroureterectomy [66]. Regardless of treatment modality, patients with low-grade lesions did well, while those with high-grade tumors were predisposed to tumor recurrence and progression.

The recommended follow-up of patients treated for upper-tract TCC should consist of interval history and physical examination, urinary cytology, and surveillance cystoscopy every 3 months for the first 2 years after treatment, every 6 months for the next 2 years and yearly thereafter if the patient is free from disease recurrence [46, 67]. Radiographic studies including chest X-ray and abdominopelvic CT should be performed every 6 months for the first 2 years and yearly thereafter. Ipsilateral endoscopy for patients who undergo organ-sparing treatment should occur every 6 months for the first 2-3 years and yearly thereafter, provided that the patient is disease free. Bone scans should only be performed for symptoms of bone pain or for an elevated alkaline phosphatase level.

## 4. The Prognostic Value of Lymphadenectomy in Patients with Muscle Invasion

Patients with muscle-invasive transitional cell carcinoma of the upper urinary tract are at high risk of nodal metastasis, and the prognosis may be extremely poor in the case of nodal involvement [68, 69].

The impact of lymph node dissection on clinical outcomes is reported only in few papers. Komatsu et al. [68] evaluated a limited cohort of 36 patients and suggested that lymph node dissection may provide a therapeutic benefit by selecting patients with lymph node metastasis as candidates for adjuvant therapy. Miyake et al. [70] reported on 72 patients with transitional cell carcinoma of the upper urinary tract. 35 of those had undergone total nephroureterectomy and regional lymphadenectomy. Lymph node dissection was associated with an increased cancer-specific survival in patients with no evidence of lymph-vascular invasion. On the other hand, in patients with evidence of lymph-vascular invasion who were considered at higher risk of micrometastatic disease, no additional prognostic advantages were provided by lymph node dissection.

In contrast, no data is available on the extent of lymph node dissection in patients with invasive transitional cell carcinoma of the upper urinary tract.

Recently, Brausi et al. [71] showed that in patients with muscle-invasive transitional cell carcinoma of the upper urinary tract, disease-free survival and cancer-specific survival were significantly higher in patients who had retroperitoneal lymph node dissection in conjunction with nephroureterectomy than in patients who did not undergo lymph node dissection. They recommended that an accurate and extended lymph node dissection can be curative in patients with advanced transitional cell carcinoma of the upper urinary tract. Nevertheless, they did not analyze the impact of the number of removed lymph nodes on clinical outcome.

Roscigno et al. [72] first tested the role of lymph node dissection on disease-free survival and cancer-specific survival. They observed 132 consecutive patients with muscle-invasive transitional cell carcinoma of the upper urinary tract who underwent radical surgery. Lymph node dissection was performed in 95 cases. Patients were stratified according to the presence of lymph node dissection and lymph node status.

They concluded that patients undergoing lymph node dissection at the time of radical surgery had a significantly better prognosis, contrary to those managed with tumor excision only, even though, in the group of patients undergoing lymph node dissection, about 1/4 (26 patients) had nodal metastases. Then, they analyzed the clinical outcome, according to nodal status. They observed that the prognosis of patients who did not receive lymph node dissection (pNx) was significantly worse than that of pN0 patients, whereas, interestingly, both disease-free survival (DFS) and cancer-specific survival (CSS) of pNx patients were comparable to those of pN+ patients. This was confirmed at multivariable analysis, where lymph node status emerged as a significant predictor of DFS and CSS after accounting for age at diagnosis, T stage, G grade, CIS (cancer in situ), LVI (lymph-vascular invasion), year of surgery and postoperative chemotherapy.

These data are in contrast with those presented by a recent paper of Brown et al. [73], showing that survival of Nx and N0 patients is similar and significantly higher than N+ patients. On the other hand, the M. D. Anderson series evaluated superficial tumor also, whereas in this series only muscle-invasive transitional cell carcinomas were considered. Probably in their population a higher percentage of pNx patients could have had positive nodes if lymph node dissections were performed.

Finally, these results suggest for the first time that the number of lymph nodes removed and examined, related to the extent of lymph node dissection, seems to play a significant role in predicting clinical outcome after radical surgery. In fact, even when only the subset of patients managed with lymph node dissection was analyzed, the number of lymph nodes removed and examined emerged, both in invariable and multivariable analyses, as a significant predictor of DFS and CSS, independently from the evidence of nodal metastases. A better clinical outcome was observed in those patients in whom at least six lymph nodes had been removed and examined [74].

## 5. Adjuvant Therapy

### 5.1. Immunotherapy

More than one third of the patients with endoscopically treated upper tract TCC will develop tumor recurrence [46]. In order to reduce recurrence rates, adjuvant topical immunotherapy or chemotherapy can be used. There are several methods to perform an instillation: by infusion through a percutaneous nephrostomy tube, via a retrograde ureteral catheter, or by retrograde reflux from the bladder with an indwelling double-J stent, or by surgical creation of ureteral reflux. The aim of the treatment is a continued exposure of the urothelium to the topical agent while maintaining a low-pressure system that is free of infection. These approaches minimize major complications such as sepsis, although granulomatous changes in the kidney and systemic adverse effects relating to bacillus Calmette-Guerin (BCG) infection can occur [75, 76].

The same agents used to treat urothelial carcinoma of the bladder can be used to treat tumorsof the upper tracts. The most common agents instilled are BCG or mitomycin-C.

As regarding the specific role of upper tract immunotherapy and topical chemotherapy, there are few reports in the literature. Thalmann et al. [77] reported on 41 renal units treated in 37 patients with BCG (Bacillus Chalmette-Guerin) via percutaneous nephrostomy tube with a mean follow-up of 44 months. For carcinoma in situ (CIS), there were treated 25 renal units and another 16 received adjuvant BCG therapy for superficial tumors in 15 patients. In this study no tumor seeding occurred along the nephrostomy tract. Indications for treatment in this study included solitary renal units, renal insufficiency, bilateral disease, and inoperable disease. Among the patients with CIS, 9 died of disease (41%), 6 died of other causes (27%), and 7 are alive at a median follow-up of 50 months (32%). Median overall survival and time to recurrence were 44 and 25 months, respectively. Fifteen patients with papillary disease of the urinary tract in 16 renal units were treated (TaG1 in 2, TaG2 in 6, TaG3 in 2, T1G3 in 2, and Tx in 4). Overall survival was 40 months (range of 1–59). Thirteen patients (87%) had recurrence after a median interval of 10 months (range of 1–69) and progression after a median interval of 11 months (range of 5–27). Among the 15 patients, 4 are alive, 6 died of disease, and 5 died of other causes with tumor present in the upper urinary tract.

The authors concluded that papillary and solid tumor recurrences of the upper urinary tract could not be prevented with BCG therapy. However, BCG therapy did provide cure in approximately 50% of renal units with CIS. Several other studies also support the use of BCG for upper tract CIS [78–83].

Vasavada et al. [84] also reported on the use of BCG in the adjuvant setting for upper tract urothelial tumor. After surgical resection for upper tract transitional cell carcinoma in eight patients, they received adjuvant BCG therapy. Grades 1, 2, and 3 diseases were present in 2, 5, and 1 patients, and Ta, T1, and T2 diseases occurred in 5, 2, and 1 patients in this cohort. At a mean follow-up of 23.8 months, 5 out of 8 patients (62.5%) were disease free, 2 out of 8 patients (25%) died of disease, and 1 out of 8 (12.5%) was alive with metastatic disease and receiving systemic chemotherapy.

Although the study number was small, the authors concluded that the application of BCG after definitive resection of the primary tumor may result in a decreased incidence of local tumor recurrence.

To our knowledge, there has not been any randomized, prospective, placebo-controlled trial specifically addressing the effectiveness of topical immunotherapy or chemotherapy for adjuvant treatment of upper tract urothelial tumors [77–85]. Until such studies become available, adjuvant therapy may be used in patients undergoing nephron-sparing management of upper tract transitional cell carcinoma with their consent and the addition of a strict surveillance protocol.

### 5.2. Radiation Therapy and Systemic Chemotherapy

The fact that the transitional cell carcinoma of the upper urinary tract is relatively rare has led to a scarcity of studies that analyze adjuvant radiation therapy and chemotherapy for locally advanced but completely resected upper tract urothelial tumors. When the tumor extents beyond the muscular, the 5-year-survival rates will be between 0% and 34% [5, 9, 86, 87]. The loco-regional recurrence at 5 years after treatment with definitive surgery, when no adjuvant chemotherapy was given, has been reported at several studies between 45% and 60% [88–90]. This high recurrence rate has been a strong argument for adjuvant therapy for all patients with locally advanced disease even after complete resection.

However, all recent studies count on limited numbers of patients because of the rarity of this disease. Some researches have supported [88, 89, 91] the role of adjuvant radiation treatment on upper tract urothelial malignancies and others have rejected it [92, 93]. The role of adjuvant chemotherapy alone for transitional cell carcinoma also remains controversial [94].

Maulard-Durdux et al. [92] reported their experience on postsurgical irradiation in 26 patients with upper tract tumors after complete resection. 11 patients had stage B disease (muscular invasion) 42% and 15 patients had stage C disease (periureteral fat invasion) 58%. According to tumor grade, 10 patients had grade 2, 40%, 15 had grade 3, 60%, and it was unknown in a patient. The radiation therapy dose was 45 Gy in all patients. After a mean follow-up of 45 months, 13 patients (50%) were alive, with 11 patients being disease free. Disease metastasized in 14 patients to the bone, liver, and lungs. The overall 5-year-survival rates and 5-year survival with no evidence of disease were 49% and 30%, respectively. The authors concluded that adjuvant radiation therapy did not improve long-term survival and is only recommended for prospective randomized studies.

A recent review of selected series of surgery with or without adjuvant radiation therapy for carcinoma of the upper urinary tract revealed some improvement in percent loco-regional failure [88–90, 92–94]. Six series of patients who received adjuvant radiation revealed a failure rate between 9% and 38%. The number of patients ranged from 9 to 45, with 1 series having 86 patients. The 5-year-survival rate was 21% to 49%. The series of patients who had surgery only (the number of patients in these series ranged from 11 to 81) without adjuvant treatment had a crude loco-regional failure rate of 45% to 65% and a 5-year-survival rate of 17% to 33%. These studies might conclude that with radiation, there seems to be some improvement in the failure and survival rate, but large studies need to be performed.

The urothelial tumors of the upper urinary tract are considered to be chemosensitive tumors [43–45]. Most of the data regarding the clinical efficacy of chemotherapy in the neoadjuvant and adjuvant settings are based on experience from bladder TCC. Advantages of neoadjuvant chemotherapy include eradication of subclinical metastatic disease, better tolerability before surgical extirpation, and ability to deliver higher doses than in the adjuvant setting [45]. Both the Advanced Bladder Cancer Meta-analysis Collaboration and the Southwest Oncology group have presented compelling data for the use of neoadjuvant platinum-based chemotherapy regimens before radical cystectomy [95, 96]. Regimens comprised of gemcitabine and cisplatin that provide a similar survival advantage to methotrexate-vinblastine-doxorubicin-cisplatin (MVAC), with a better safety profile and tolerability, increase the attractiveness of neoadjuvant chemotherapy [97]. Similar management strategies are likely to be beneficial for upper-tract TCC, particularly in the setting of large, bulky tumors.

The role of adjuvant systemic chemotherapy to patients with locally advanced upper urinary tract tumors is not well defined, because of the scarcity of controlled trials due to the low prevalence and incidence of disease. Nevertheless, a recent study by Brown et al. [73] reported on their experience with both adjuvant radiation therapy and concurrent chemotherapy for locally advanced disease. After surgery, 31 patients have received adjuvant radiation therapy. All patients had grade 2 and even 84% of the group were found to have a pathologic stage of T3 or higher. Nine patients received methotrexate, cisplatin, and vinblastine chemotherapy for 2 to 4 cycles. Univariate analysis revealed that patients had improved 5-year actuarial overall and disease-specific survival with the administration of concurrent chemotherapy when compared with patients receiving adjuvant radiation alone (27% versus 67%, *P* = .01; 41% versus 76%, *P* = .06, resp.).

## 6. Conclusions

Treatment of upper-tract urothelial carcinoma has developed and changed with advances in technology. Treatment has evolved from open radical nephroureterectomy to percutaneous resection to ureteroscopic treatment. Adjuvant treatments are also evolving with topical immunotherapy, radiation, and chemotherapy. Before any decision for optimal treatment, the specifics of each individual patient with regard to renal function, medical comorbidities, location of disease, tumor stage, and tumor grade must be taken into account.

Due to the fact that the incidence and prevalence of this tumor is low, the majority of series in the literature are of limited number. What is clear from the literature with regard to surgical outcomes for upper-tract TCC is that this is a potentially lethal disease if not treated appropriately. Due to its relative rarity, many decisions regarding treatment are extrapolated from our experience in managing bladder urothelial carcinoma (such as node dissections, topical chemotherapies, immunotherapies, and adjuvant treatments). The problem about the studies utilizing minimally invasive techniques is that they lack long-term follow-up. Almost all of the studies are retrospective in nature and therefore flawed with selection biases.

As a result, the standard way still remains to be surgical removal with radical nephroureterectomy, and for selected patients segmental ureterectomy may be performed. Endoscopic management is also reasonable in patients with low-grade and low-stage disease as long as they adhere to a strict follow-up protocol that includes frequent cytology and endoscopy. The benefits of adjuvant radiation and chemotherapy are still debated, but the literature does reveal some improvement in disease-specific survival using both forms of treatment.

## Figures and Tables

**Table 1:** Studies that compare laparoscopic nephroureterectomy with open surgery.

Study	No. of patients	Tumor grade	Follow-up	Outcomes
Gill et al. [12]	42 in LT, 35 in OS	34 patients in LT arm and 28 in OS arm III tumors	11.1 months in LT,34.4 in OS	LT significantly decreasing morbidity with comparable oncological and survival data to OS

Shalhav et al. [13]	25 in LT 17 in OS	21 patients in LT arm and 14 in OS arm grade II	2 years in both arms	LT has longer operating time but the same efficacy and is better tolerated

LT: laparoscopic treatment, OS: open surgery.

**Table 2:** Studies that compare hand-assisted laparoscopic nephroureterectomy with other techniques.

Study	No. of patients	Tumor grade	Follow-up	Outcomes
Kawauchi et al. [18]	34 in HALN 34 in OS	24 patients in HALN arm and 25 in OS arm grade II	13.1 months in HALN,48.8 in OS	TTR for HALN 9.5 months with RR 12% TTR for OS 14.4 months with RR 47%
Seifman et al. [26]	16 in HALN 11 in OS	12 patients in HALN arm and 9 in OS grade II tumors	19 months in HALN,16 in OS	TR for HALN in 3 patients and for OS in 7 patients
Landman et al. [20]	16 in HALN 11 in LN	13 patients in HALN arm and 8 in LN arm grade III tumors	9.6 months in HALN,27.4 in LN	HALN decreases operative time without significantly altering short-term parameters of convalescence

HALN: hand-assisted laparoscopic nephroureterectomy, LN: laparoscopic nephroureterectomy, OS: open surgery, TTR: time to recurrence, RR: recurrence rate, TR: tumor recurrence.

**Table 3:** Studies for robotic-assisted laparoscopic nephrectomy.

Study	No. of patients	Conversions	Follow-up	Outcomes
Gettman et al. [28]	13	1 to LN	13 months	RALN is feasible and safely performed
Phillips et al. [30]	12	2 one to HALN and 1 to OS	12 months	RALN is safe, feasible, and reproducible
Caruso et al. [31]	10	1 to LN	12 months	RALN safe and feasible procedure in patients with small exophytic masses
Kaul et al. [32]	10	No conversions	15 months	RALN is a viable alternative to LN for patients with small exophytic masses
Deane et al. [33]	10	No conversions	16 months	No difference between RALN and LN as regarding PT,IBL and MWIT

RALN: robotic-assisted laparoscopic nephrectomy, LN: laparoscopic nephrectomy, OS: open surgery, PT: procedure time, IBL: intraoperative blood loss, MWIT: mean warm ischemia time.

**Table 4:** Studies that compare techniques for the management of distal ureter end bladder cuff with other methods of treatment.

Study	No. of patients	Tumor grade	Follow-up	Outcomes
*Open technique*				

Matsui et al. [22]	17 in OT 17 in SN	14 patients OT arm and 13 in SN arm grade III	8.8 months in OT and 23 months in SN	No difference in DFS
Klingler et al. [16]	19 in OT 15 in SN	15 patients in OT and 13 in SN arm grade II	21.1 months in OT and 23.1 months in SN	CCR and RTR similar in both arms

*Transvesical laparoscopic technique*				

Gill et al. [12]	42 in LT 35 in SN	34 patients in LT arm and 28 in OS arm III tumors	11.1 in LT and 34.4 months in SN	CSS and TR comparable in both arms

*Laparoscopic stapling technique*				

Shalhav et al. [13]	25 in LT 17 in SN	21 patients in LT arm and 14 in OS arm grade II	24 months LT 43 months SN	RTR lower in LT

*The “pluck” technique*				

McNeill et al. [36]	25 in PT 42 in SN	18 patients in PT arm grade II and 36 patients in SN arm grade III	32.9 months PT 42.3 months SN	No difference in TR

SN: standard nephroureterectomy, OT: open technique, CCR: cancer control rate, RTR: risk of tumor recurrence, CSS: cancer specific survival, TR: tumor recurrence, ORN: open radical nephroureterectomy, PT: “pluck” technique.
